# Species composition and elevational distribution of bumble bees (Hymenoptera, Apidae, *Bombus* Latreille) in the East Himalaya, Arunachal Pradesh, India

**DOI:** 10.3897/zookeys.851.32956

**Published:** 2019-06-03

**Authors:** Martin Streinzer, Jharna Chakravorty, Johann Neumayer, Karsing Megu, Jaya Narah, Thomas Schmitt, Himender Bharti, Johannes Spaethe, Axel Brockmann

**Affiliations:** 1 Department of Neurobiology, Faculty of Life Sciences, University of Vienna, Althanstraße 14, 1090 Vienna, Austria University of Vienna Vienna Austria; 2 Department of Zoology, Rajiv Gandhi University, Rono Hills, Doimukh, Papum Pare, Arunachal Pradesh 791112, India Rajiv Gandhi University Papum Pare India; 3 Obergrubstraße 18, 5161 Elixhausen, Austria Unaffiliated Elixhausen Austria; 4 National Centre for Biological Sciences, Tata Institute of Fundamental Research, Bellary Road, Bengaluru 560065, Karnataka, India Tata Institute of Fundamental Research Bangalore India; 5 Department of Animal Ecology and Tropical Biology (Zoology III), Biocenter, University of Würzburg, Am Hubland, 97074 Würzburg, Germany University of Würzburg Würzburg Germany; 6 Department of Zoology and Environmental Sciences, Punjabi University, Patiala, Punjab 147002, India Punjabi University Patiala India; 7 Department of Behavioral Physiology and Sociobiology (Zoology II), Biocenter, University of Würzburg, Am Hubland, 97074 Würzburg, Germany National Centre for Biological Sciences, Tata Institute of Fundamental Research Bangalore India

**Keywords:** Alpine habitats, Apidae, conservation, global change, insect collection, pollination

## Abstract

The East Himalaya is one of the world’s most biodiverse ecosystems. However, very little is known about the abundance and distribution of many plant and animal taxa in this region. Bumble bees are a group of cold-adapted and high elevation insects that fulfil an important ecological and economical function as pollinators of wild and agricultural flowering plants and crops. The Himalayan mountain range provides ample suitable habitats for bumble bees. Systematic study of Himalayan bumble bees began a few decades ago and the main focus has centred on the western region, while the eastern part of the mountain range has received little attention and only a few species have been verified. During a three-year survey, more than 700 bumble bee specimens of 21 species were collected in Arunachal Pradesh, the largest of the north-eastern states of India. The material included a range of species that were previously known from a limited number of collected specimens, which highlights the unique character of the East Himalayan ecosystem. Our results are an important first step towards a future assessment of species distribution, threat, and conservation. Clear elevation patterns of species diversity were observed, which raise important questions about the functional adaptations that allow bumble bees to thrive in this particularly moist region in the East Himalaya.

## Introduction

Bumble bees (Hymenoptera, Apidae, *Bombus* Latreille) are a group of conspicuous, large and colourful bees that mainly inhabit cold and temperate habitats at high latitudes and elevations. Their conspicuous appearance and abundance established them a prime object of study for numerous early naturalists and insect collectors. After extensive revision in the past decades, around 260 species are currently recognized (Williams 1998; updated online at http://www.nhm.ac.uk/research-curation/research/projects/bombus/index.html).

Current global sampling efforts focus on revising the bumble bee taxonomy at the subgeneric level and filling in blank spots in global distribution data for a worldwide IUCN red list assessment of all species (http://iucn.org/bumblebees). The latter is urgently needed, since a number of bumble bee species have recently shown dramatic declines in their abundance and range (Cameron et al. 2011). The reasons are only partially understood and most likely involve pathogen spill-over from commercial breeding and changes in agricultural practices and land use (Cameron et al. 2011, Jacobson et al. 2018). Moreover, climate change poses a threat to many bumble bee species worldwide, especially those adapted to high elevations, due to an ongoing decline of suitable habitats (Hoiss et al. 2012, Kerr et al. 2015, Rasmont et al. 2015).

Bumble bees are pollinators of many wild flowers. They are abundant throughout the season and, due to their thermoregulatory abilities, are able to be active at very low ambient temperatures (Corbet et al. 1993). Thus, they serve as important pollinators, especially in alpine environments and early in the flowering season (Kevan and Baker 1983, Yu et al. 2012). Besides their ecological importance, bumble bees serve as pollinators for many cultivated fruits, vegetables and spices, and thus become economically valuable, as well. In the industrialized western world, more than one million colonies per year are commercially reared and sold for pollination purposes (Velthuis and van Doorn 2006).

Bumble bees are cold adapted and therefore are most diverse and abundant in northern temperate habitats and in alpine environments. The Himalaya, the longest mountain range in the world, is home to a high bumble bee diversity due to its variety of suitable habitats. The mountain range spreads over 3,000 km between the Karakorum in the west and the Patkai and Hengduan mountain ranges in the east. As a major barrier for the south-eastern monsoon winds, it plays an important role in shaping the climate of entire South Asia (Zhisheng et al. 2001, Xu et al. 2009). The climate in the Himalaya is particularly diverse, e.g., the western end shows strong annual temperature fluctuations and is relatively arid whereas the eastern end is rather stable in the annual temperatures and receives a high amount of annual rainfall. These climatic differences account for distinct differences in flora and fauna (Williams et al. 2010, Rawat 2017). The West Himalaya is characterized by temperate broad leaf forests and arid alpine meadows and pastures at high elevations with relatively low annual rainfall (Rawat 2017). At the eastern end, in contrast, annual precipitation can reach up to 5,000 mm (Dhar and Nandargi 2006) allowing for the formation of subtropical broadleaf forests and moist alpine meadows at higher elevations (Rawat 2017). Previous studies found that the biodiversity in the East Himalaya is particularly rich and the region is considered a global hotspot of biodiversity (Myers et al. 2000).

So far, bumble bee composition was intensively studied in the West (Williams 1991, Saini et al. 2015) and Central Himalaya (Williams et al. 2010). The highest diversity is reported for the Central Himalaya, i.e., from Nepal and the Indian state of Sikkim (Williams 2004, Williams et al. 2010, Saini et al. 2015). Many eastern and western species reach their respective distribution limit in Nepal and the overlap of both faunal regions may contribute to the high bumble bee diversity in this area (Williams et al. 2010). The eastern end of the Himalayan mountain range has received little attention so far and only few actually confirmed records are available (Williams 2004, Saini et al. 2015). The inaccessibility and the harsh climatic conditions cause field work in the East Himalaya to be extremely challenging (see comments in Saini et al. 2015, Rawat 2017) and has certainly contributed to the lack of bumble bee research. Arunachal Pradesh, the northernmost and largest of the Indian northeast region (NER) states, comprises the eastern end of the Himalayan range. Arunachal Pradesh is unique, in that it is densely forested, sparsely populated and agriculturally only extensively managed and thus barely fragmented in its landscape (Tripathi et al. 2016). Previous studies also showed an outstanding biodiversity and high endemism, e.g., in *Rhododendron* species, bamboos, orchids and many other plant taxa (Bhuyan et al. 2003, Mao 2010, Paul et al. 2010, Rawat 2017) as well as butterflies (Sondhi and Kunte 2016).

In this study, the results from the first systematic survey of bumble bees in Arunachal Pradesh are reported based on material collected during three major and a few minor field trips during the years 2015–2017. The survey represents the first phase of a project aiming to (1) document the bumble bee diversity in the East Himalaya to aid global distribution range assessments, (2) identify local pollinators of fruits, vegetables and other crops, and (3) describe functional adaptations that allow bumble bees to thrive in the particularly challenging climate of the East Himalaya.

## Materials and methods

### Study area and locations

**Figure 1.**
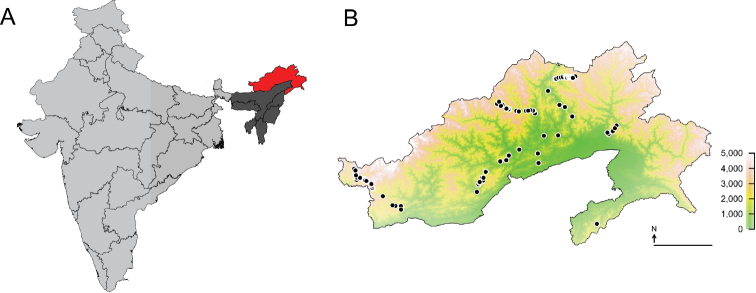
Sampling locations **A** Mainland India (light grey) showing the geographic location of Arunachal Pradesh (red) in the northeast region (NER, dark grey). Outlines denote Indian state borders. **B** Sampling locations within the state of Arunachal Pradesh for three major and a few minor field trips between 2015 and 2017. The locations are projected from GPS data to a SRTM elevation data set. The colour scale refers to elevation and does not reflect vegetation zone. Scale in B represents 100 km.

Arunachal Pradesh is the largest of the North-East Indian states and is bordered by Bhutan in the west, the People’s Republic of China (Autonomous region of Tibet) in the north, Myanmar in the east and the Indian states of Assam and Nagaland in the south (Fig. 1A).

**Figure 2.**
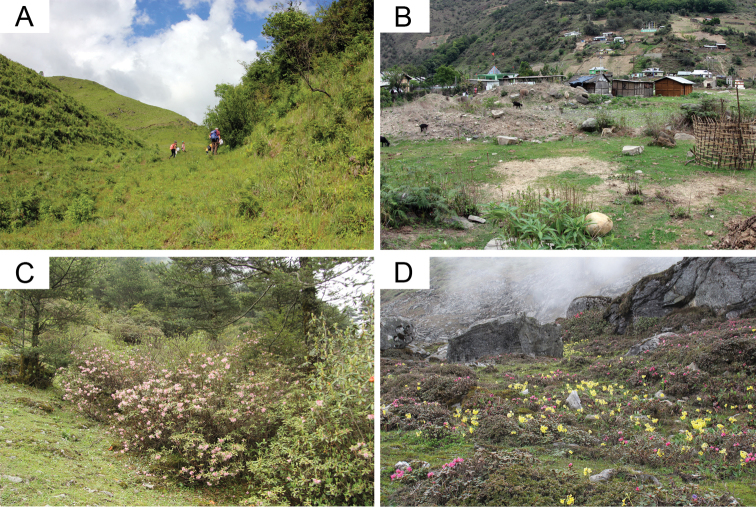
Bumble bee habitats in Arunachal Pradesh **A** Grass–/shrubland at 1,950–2,050 m elevation (Mechuka, West Siang district). Workers of *B.festivus* and *B.luteipes* and workers and males of *B.flavescens* were observed visiting *Cotoneaster* bushes **B** Agricultural crops located in a river valley at 1,500 m elevation (Old Dirang, West Kameng district). Workers of *B.flavescens* were collected from *Punicagranatum* flowers **C** Ever-green deciduous *Rhododendron*– and *Pinus*-forests at 3,500 m (Karpo, Tawang district), where we collected queens of *B.festivus* and *B.pressus***D** Alpine meadow with flowering *Primula* sp. and *Rhododendron* sp. (Se-La Pass, Tawang district) at 4,260 m, where we collected *B.mirus*, *B.lemniscatus*, *B.nobilis*, *B.festivus*, *B.rufofasciatus*, *B.miniatus*, and *B.novus*.

Bumble bee specimens were collected during three major field surveys in the years 2015–2017. The field trips covered the entire flowering season, pre-monsoon (May–Jun. 2016), during monsoon (Aug.–Sep. 2017), and post-monsoon (Sep.–Oct. 2015). Additional specimens were collected from the entire state during shorter field visits (post-monsoon) in the years 2016–2017 (Fig. 1B). We covered elevations between ca. 200 m and ca. 4,300 m above sea level and habitats ranging from foothill forests (tropical wet evergreen and semi-evergreen), temperate broadleaf forest, subalpine forest up to the alpine zone (Fig. 2; Rawat 2017). GPS locations and elevations were recorded using handheld GPS units or cell phones (Garmin Ltd., CH; Apple Inc., CA, USA) and later verified using Google Earth Pro (version 7.3.2, Google LLC, CA, USA). Elevation was read from the GPS unit and rounded to the closest 10 m for the analysis. Mapping of the occurrence data was performed using GPS coordinates and SRTM digital elevation data (Jarvis et al. 2008) using the “Raster” package (build 2.6-7; Hijmans 2017) in R (build 3.5.1; R Core Team 2018).

### Sample collection

Bumble bees were collected by sweep netting and immediately killed with cyanide or ethyl acetate. The specimens were then stored in airtight containers with a few layers of tissue and the addition of a few drops of ethyl acetate to prevent the growth of mould during transport. After the field sampling, specimens were dry-mounted on standard insect pins for identification. The collected specimens were deposited in the NCBS Research Collection (National Centre for Biological Sciences, Tata Institute of Fundamental Research, Bangalore, India) for future reference. A full list of the collecting information of the museum specimens is available upon request (curators: Dr Axel Brockmann and Dr Krushnamegh Kunte, NCBS Bangalore). In addition to the collected specimens, some field observations were conducted. Since the observed specimens are not available for later reference, only those are included that could be unambiguously identified and that were from locations where additional voucher specimens of the same species were collected. In addition to the specimens collected in this project, entomological collections were examined for bumble bees from Arunachal Pradesh.

### Experimental ethics

Permits to sample bumble bees were issued by the Government of Arunachal Pradesh to Jharna Chakravorty (No. SFRI/APBB/9/2011-846, No. SFRI/APBB/09/2016/1168) and to Himender Bharti (No. CWL/G/13 (95)/2011-12/Pt./2471-75).

### Species identification

Specimens were identified using published identification keys for adjacent regions, e.g., Kashmir (Williams 1991), Nepal (Williams et al. 2010), Sichuan (Williams et al. 2009), North China (An et al. 2014), and India (Saini et al. 2015). In addition, first descriptions and detailed species accounts were consulted (Linnaeus 1758, Smith 1852a, 1861, 1852b, Bingham 1897, Friese 1905, 1916, 1918, Skorikov 1912, Frison 1933, 1935, Richards 1934, Tkalcu 1968a, 1974).

## Results

Between 2015 and 2017, 773 bumble bee specimens were either collected, identified in the field and from photographs or identified in entomological collections (Table 1). A total of 642 specimens were deposited in the NCBS Research Collection. The remaining voucher specimens are part of research project voucher collections (coll. Jaya Narah, Department of Zoology, Rajiv Gandhi University, Itanagar, Arunachal Pradesh – 15 specimens). An additional 16 specimens (collected 2014– 2017) were identified in entomological collections (Department of Entomology, University of Agricultural Sciences, GKVK, Bangalore – 15 specimens, India; NBCS Research Collection, Bangalore, India – 1 specimen).

**Figure 3.**
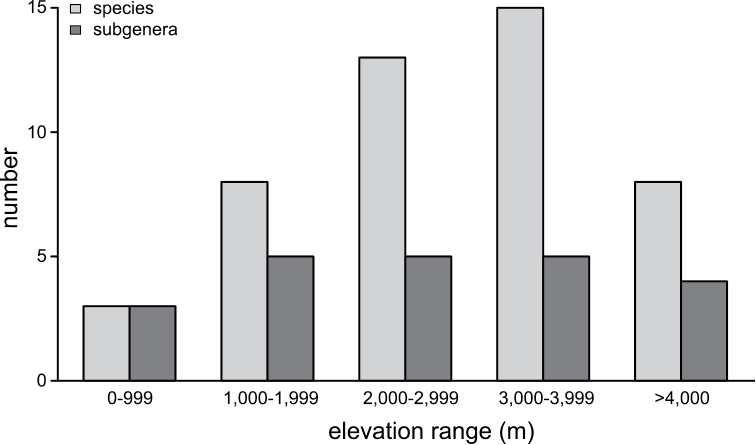
Species and subgeneric diversity along the elevational gradient. In the lowland tropical forest (< 1,000 m) only *B.haemorrhoidalis*, *B.albopleuralis*, and *B.breviceps* were observed. With increasing elevation, we found an increasing diversity of species. The relatively low diversity at > 4,000 m may represent a sampling bias, since only a few locations were accessible.

The sampled region covers most of the state Arunachal Pradesh, and the least amount of sampling was carried out in the eastern-most region (Fig. 1B). Bumble bees were collected in a large elevation range from 230 m to 4,260 m above sea level, covering many different habitat types (Fig. 2). There was a clear elevational change in species composition (Fig. 3). In the moist evergreen forest at low elevations (230–1,090 m), only three species from three different subgenera were observed (B. (Orientalibombus) haemorrhoidalis Smith, B. (Megabombus) albopleuralis Friese, B. (Alpigenobombus) breviceps Smith; Table 1, Fig. 3, Suppl. material 1, Figs S1B, 1C, 1N). Species diversity increased with elevation, climaxing in the region 3,000–4,000 m (mostly corresponding to the subalpine stage) with 15 species from five subgenera (Fig. 3). In total, the collected specimens belong to 21 currently recognized species from six subgenera (Table 1).

**Table 1.** Summary of the collected bumble bee specimens. All specimens (N = 773) are listed that were examined and identified by the authors, including material collected during the field trips, specimens from research and museum collections, and specimens identified in the field. Subgenera are sorted according to their phylogenetic position (Williams et al. 2008). Within the subgenera, species are sorted alphabetically. Elevation has been rounded to the closest 10 m. Abbreviations: **Q** – number of queens, **W** –number of workers, **M** – number of males, † – includes one specimen of unspecified location or imprecise locality information.

**Subgenus**	**Species**	**Σ**	**Q**	**W**	**M**	**Elevation**	**No. Localities**
*** Orientalibombus ***	*B.funerarius* Smith,1852	3	0	2	1	2,400–3,230	2
	*B.haemorrhoidalis* Smith,1852	150	13	130	7	400–3,450	48†
*** Megabombus ***	*B.albopleuralis* Friese,1916	83	5	70	8	230–2,990	40†
*** Psithyrus ***	*B.cornutus* (Frison,1933)	1	0	NA	1	3,280	1
	*B.novus* (Frison,1933)	1	1	NA	0	4,200	1
*** Pyrobombus ***	*B.abnormis* (Tkalcu,1968)	4	4	0	0	3,680–3,940	2
	*B.flavescens* Smith,1852	31	2	22	7	1,510–3,130	8
	*B.hypnorum* s.l. (L.,1758)	9	0	4	5	2,850–3,980	5
	*B.lemniscatus* Skorikov,1912	10	6	1	3	3,500–4,260	5
	*B.luteipes* Richards,1934	76	0	70	6	1,150–3,500	21
	*B.mirus* (Tkalcu,1968)	98	17	51	30	2,850–4,260	24
	*B.parthenius* Richards,1934	20	0	16	4	2,950–3,680	8
	*B.pressus* (Frison,1935)	41	4	27	10	3,510–4,030	19
*** Alpigenobombus ***	*B.breviceps* Smith,1852	34	3	28	3	480–2,790	19
	*B.genalis* Friese,1918	6	0	6	0	1,560–1,850	3
	*B.grahami* (Frison,1933)	2	0	2	0	2,710	1
	*B.nobilis* Friese,1905	75	4	61	10	3,780–4,260	21
*** Melanobombus ***	*B.eximius* Smith,1852	9	1	8	0	1,090–1,720	6
	*B.festivus* Smith,1861	63	4	54	5	1,940–4,260	21
	*B.miniatus* Bingham,1897	31	1	17	13	2,400–4,240	11
	*B.rufofasciatus* Smith,1852	26	11	11	4	2,400–4,260	11

## Discussion

### Bumble bee diversity and species records in the East Himalaya

During several field trips in the Indian state of Arunachal Pradesh, over 700 bumble bee specimens were collected, belonging to 21 species. This survey represents the first systematic study of bumble bee diversity in the East Himalayan range, an area known as a biodiversity hotspot and an important region for conservation priority (Myers et al. 2000).

Previously, extremely few confirmed records for *Bombus* exist for Arunachal Pradesh. Williams (2004) listed eight species and predicted the occurrence of another 13 based on their known distribution. During a 12 year survey of India, and based on a total of almost 7,000 specimens, Saini et al. (2015) only recorded a single species, B. (Melanobombus) eximius Smith, from this state. In the present study, individuals of 21 currently recognized species were collected (Table 1), including almost all of the previously confirmed species (except for B. (Psithyrus) turneri (Richards)) and more than half of the predicted species (Williams 2004). Furthermore, a number of the species collected were previously assumed to either have a West Himalayan, e.g., B. (Melanobombus) miniatus Bingham, B. (Psithyrus) novus (Frison), B. (Pyrobombus) parthenius Richards, or Central Himalayan distribution, e.g., B. (Pyrobombus) abnormis (Tkalcu), B. (Pyrobombus) mirus (Tkalcu), B. (Pyrobombus) pressus (Frison), and were not expected to occur in Arunachal Pradesh (Williams 2004). Many of these species were previously classified as vulnerable, near threatened (Williams & Osborne, 2009) or extremely rare (Saini et al. 2015), are known from a limited number of specimens in entomological collections (PH Williams, personal communication, July 2018), and could not be found in recent field surveys across India (Saini et al. 2015). *Bombusmirus*, a species previously considered confined and rare (Tkalcu 1968a, Williams et al. 2010, Saini et al. 2015) represented ~13% of our entire collection (Table 1).

The present checklist for Arunachal Pradesh, comprising 22 species (including *B.turneri*, which was not found in our survey), places Arunachal Pradesh close to the species diversity found in the West Himalaya, e.g., Kashmir [29 species], Himachal Pradesh [25] and Uttarakhand [22] (Williams 2004, Williams et al. 2010). Contrary to the East Himalaya, these regions were intensively sampled in the last decades (Williams 1991, Saini et al. 2015). Based on the current sampling status and the predictions by Williams (2004), additional species are expected to be found in the future. Alpine regions above the tree line (> 4,000 m) are scarce and not easily accessible in Arunachal Pradesh (Mishra et al. 2006). A more intense survey of these areas will possibly confirm the presence of high elevation species (e.g., *B.waltoni* Cockerell, *B.kashmirensis* Friese, *B.ladakhensis* Richards, *B.keriensis* Morawitz), which are known to occur in south-east Tibet close to the Indian border (Williams 2004, Williams et al. 2015). The East Himalayan region is still vastly under-sampled and more thorough sampling is needed in the entire NER of India at the intersection between the Himalaya and the Patkai mountain range and in the mountain regions of Meghalaya, where the general occurrence of bumble bees is confirmed, but systematic surveys are lacking (Frison 1933, Tkalcu 1974, 1989, Williams 2004, Saini et al. 2015).

Future work in the region will also provide material for taxonomic revisions. Resulting from the large number of specific, subspecific, and infrasubspecific synonyms, a genus wide revision is still under progress (Williams 1998). The treatment by Saini et al. (2015) had not incorporated recent taxonomic changes from sub-generic revisions (e.g., Williams et al. 2011, 2012). While the identity of many species in our study is clear from the morphology, a few nominal taxa are currently treated as belonging to a species complex and future work will likely elucidate their taxonomic treatment (e.g., *B.hypnorum* s. l. (L.); see Tkalcu 1974, Williams et al. 2010).

### Mimetic circles

Particularly high local convergence in colour pattern is often found within the genus *Bombus*. It is usually interpreted as Müllerian mimicry (Richards 1929, Williams 2007). One of the most remarkable mimetic circles is found in the Himalaya and South-East Asia, comprising B. (Orientalibombus) haemorrhoidalis, B. (Alpigenobombus) breviceps, B. (Pyrobombus) rotundiceps Friese and the closely related species of the B. (Megabombus) trifasciatus-group (Tkalcu 1968b, Williams 1991, Hines and Williams 2012). The species are members of four different subgenera, corroborating the interpretation that convergent evolution, rather than common ancestry, is responsible for the similarity of the colour pattern.

**Figure 4.**
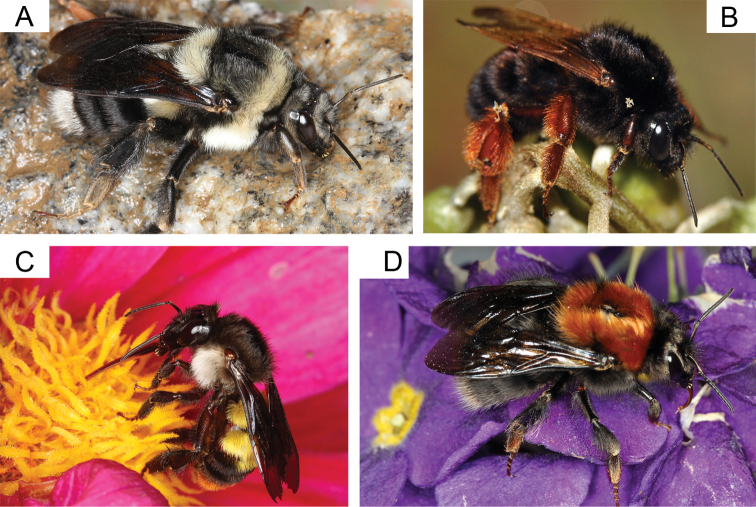
Examples of bumble bee species collected in Arunachal Pradesh **A***Bombusminiatus* (queen) is a West Himalayan species of the subgenus Melanobombus reaching its eastern distribution limit in Arunachal Pradesh **B***Bombusgenalis* (worker), a rare species of the mid-elevation narrowly distributed in the East Himalaya **C***Bombusalbopleuralis* (worker), a widespread Himalayan species that occurs in a large elevational range from the tropical lowlands to the subalpine zone in Arunachal Pradesh **D***Bombusabnormis* (queen), an elusive and very rare high elevation species of the subgenus Pyrobombus narrowly distributed in the East Himalaya.

Three of these species were found in our study area and showed identical colour pattern across Arunachal Pradesh. Two other mimetic groups are present in the region, each comprising members of at least two different subgenera. First, B. (Pyrobombus) abnormis, B. (Pyrobombus) hypnorum s.l. and workers of B. (Melanobombus) festivus Smith all have a brown thorax and a white tail. The second group comprises B. (Pyrobombus) flavescens Smith, B. (Melanobombus) eximius and B. (Alpigenobombus) genalis Friese, which are characterized by black body pile, orange tinted wings and orange-brown cuticle and hairs on the legs (see examples in Fig. 4).

Colour pattern convergence within *Bombus* is also often observed between the parasitic species of the subgenus Psithyrus and their preferred host species (Reinig 1935, Williams 2008). The parasitic *B.novus*, recorded in our study, was previously assumed to develop in nests of B. (Melanobombus) rufofasciatus Smith (Tkalcu 1974). Although the female of *B.novus* shares with *B.rufofasciatus* a reddish band of pile just anterior to the white tail, it more closely resembles *B.miniatus* in the pale yellow (rather than white-grey) colouration of the anterior pale bands and the darker tint of the wings (Williams et al. 2010; Suppl. material 2, Fig. S2). Furthermore, the known distribution ranges of the latter match more closely, since both are (mostly) West Himalayan species that reach their eastern distribution limit in Arunachal Pradesh, whereas *B.rufofasciatus* is a widespread Himalayan and Tibetan species (Williams et al. 2015). However, most *Psithyrus* are to some extent flexible in their host choice and more observations, especially from breeding *Psithyrus* in their host nests, are necessary to confirm this suggested parasite-host association (Williams 2008).

### Elevational distribution and adaptation

Covering a large range of elevations and habitat types, clear patterns of species-specific elevational ranges were recognised (Fig. 3). A number of species were only found in the subalpine and alpine region at the highest elevations, and they occupied similar elevational niches as in other regions of the world (e.g., *B.abnormis*, B. (Pyrobombus) lemniscatus Skorikov, *B.mirus*, B. (Alpigenobombus) nobilis Friese, *B.pressus*; Williams et al. 2009, 2010). The highest species diversity was observed at elevations between 3,000–4,000 m (Fig. 3), similar to observation in the Central Himalaya (Williams et al. 2010). However, at the current stage of study, this may also represent a sampling bias from the relatively lower number of sampling points at high elevations. In general, species diversity was found to decline towards lower elevations, and in the lowland (<1,000 m) only three species (*B.haemorrhoidalis*, *B.albopleuralis*, *B.breviceps*) were found. These species also occur at relatively low elevations throughout the Himalaya (lowest elevations: *B.haemorrhoidalis*: Kashmir—1,000 m, Nepal—850 m, *B.albopleuralis*: Kashmir—1,000 m, Nepal—950 m, *B.breviceps*: Nepal—980 m; Williams 1991, Williams et al. 2010). However, our records (*B.haemorrhoidalis*—400m, *B.albopleuralis*—230 m, *B.breviceps*—480 m; see Table 1), represent the lowest elevations at which these species, and bumble bees in general, have ever been recorded in the Himalayan range (Williams 1991, Williams et al. 2010). Bumble bees often occur in a wide elevational range, but only few species reach the tropical lowland, where conditions are usually unfavourable for these cold-adapted bees (Moure and Sakagami 1962, Williams 1991, Gonzalez et al. 2004, Williams et al. 2009).

Our observations may have multiple, not mutually exclusive, explanations. First, the specific climate of the East Himalaya probably allows certain bumble bee species to thrive at relatively lower elevations (see below). Indeed, there seems to be a gradual decrease in the lower elevation limit from the west to the east that supports this interpretation (Williams 1991, Williams et al. 2010). Second, bumble bee workers can cover large horizontal and, particularly in steep terrain, vertical distances during their foraging trips (Osborne et al. 1999). In Arunachal Pradesh, most of the valleys are particularly steep and both lowland and higher elevations are within the foraging distance of a few kilometres. Therefore, the low records may represent foraging workers from a nest at higher elevation.

*B.haemorrhoidalis*, *B.albopleuralis*, and *B.breviceps* cover a wide range of elevations and usually were most abundant at medium elevations (Table 1, Suppl. material 1, Figs S1B, S1C, S1N). Nevertheless, the wide range of foraging habitats, each posing their own challenges with respect to thermoregulation and energy expenditure, is remarkable. Future work is necessary to assess their specific individual and population-level adaptations that provide the plasticity to cover such a diversity in elevations and habitat types, while other species are restricted to narrow ranges and specific habitats (Williams et al. 2009, 2010, 2018). This plasticity (or absence of it) is of particular interest when we seek to understand potential threats due to climate change, making some species more vulnerable than others.

Several physiological and behavioural adaptations have been discussed in the context of elevational adaptation in bumble bees and previous work shows that behavioural plasticity enables quick adaptation to different elevations (Dillon et al. 2006, Dillon and Dudley 2014). At the morphological and physiological level, wing load and wing aspect ratio (Cartar 1992), variation of the cuticular hydrocarbon composition, which prevents bees from desiccation (Foley and Telonis-Scott 2010, Menzel et al. 2017), or changes in mitochondrial density and/or enzyme composition (Harrison et al. 2006, Zhang et al. 2013) may be important factors that vary among populations. However, the specific adaptations that allow these species to thrive in the particularly challenging habitats in the East Himalaya, where the peak of the monsoon season coincides with the peak of colony development in many species, is subject to future investigations. Our survey identified *B.haemorrhoidalis* and *B.albopleuralis* as suitable model taxa to investigate the potential adaptations to specific climatic conditions at the individual and population level. Both species cover a wide range of elevations and are widely distributed in Arunachal Pradesh (Table 1, Suppl. material 1, Figs S1B, S1D).

### Current and Future Threats and Conservation

The discovery of many rare and confined species of bumble bees in Arunachal Pradesh highlights the importance of extensive sampling in remote regions to better understand species distribution and ecological requirements (see also the discussion in Williams 2018). Although many species may be rare or confined to a particular region from a global perspective, they can be locally abundant and/or restricted to a very specific habitat. The specific climate of the East Himalaya, with the high amount of precipitation, supports a high biodiversity including a large amount of endemism in the region (Myers et al. 2000, Mao 2010). Our observations suggest that some bumble bee species may be particularly adapted to these conditions since they are restricted to a limited region in the East Himalaya (e.g., *B.mirus*, *B.genalis*).

Arunachal Pradesh can currently be considered a remote region without serious recent land use changes, only small-scale agriculture and a low population density (Sikri 2006). However, locally distributed species and high elevation specialists may still be under future threat of extinction, due to changes in agricultural practices or climate change (Xu et al. 2009, Hoiss et al. 2012). Rising temperatures force bumble bee species to shift to higher elevations (Kerr et al. 2015), but high elevation refuges may be limited for species that are adapted to the East Himalayan climate. It is therefore crucial to better understand the adaptations of the local bumble bee fauna to assess their future threat status. Furthermore, it is urgent to develop general strategies for the future to preserve much of this remarkable region (Myers et al. 2000, Government of Arunachal Pradesh 2011).

In the Himalaya, bumble bees serve as important pollinators of many fruits, vegetables, e.g., cardamom (Deka et al. 2011), apple, and other crops (Raj et al. 2012, Raj and Mattu 2014, Tayeng and Gogoi 2018). Understanding their ecological requirements and preserving habitats that support pollinator diversity are crucial for a sufficient agricultural yield, especially in the extensively managed smallholder farming systems that are abundant in Arunachal Pradesh (Kala 2005). Bumble bees are used worldwide as pollinators for commercial fruit and vegetable production (Velthuis and van Doorn 2006). Initially, commercially reared species were used outside their native range, resulting both in the introduction of alien species (Morales et al. 2013) and spread of pathogens to native bumble bee populations (Arbetman et al. 2013). Nowadays, attempts are made to select suitable native species and develop methods for their commercial rearing in many world regions (Padilla et al. 2017). Laboratory rearing of *B.haemorrhoidalis* in India (Chauhan et al. 2014) and *B.breviceps* in Vietnam (Thai and Van Toan 2018) are first steps to produce native bumble bee colonies for commercial pollination. Both species are widespread in Arunachal Pradesh and would make excellent pollinators for many fruit and vegetables (Deka et al. 2011). Additional work is necessary to confirm their potential, or find other promising species for the future development of commercial fruit and crop pollination in Arunachal Pradesh.

## Supplementary materials

Supplementary material 1 Distribution maps of all collected bumble bee species Data type: occurrence Explanation note: Distribution maps were created from projecting GPS collection data to an SRTM elevation dataset. The colour scale represents elevation and does not reflect vegetation zone. Each map is accompanied with a histogram of the elevations at which we collected the specimen. Species grouping follows Table 1 of the main text. Scale bar represents 100 km. This dataset is made available under the Open Database License (http://opendatacommons.org/licenses/odbl/1.0/). The Open Database License (ODbL) is a license agreement intended to allow users to freely share, modify, and use this Dataset while maintaining this same freedom for others, provided that the original source and author(s) are credited.Martin Streinzer, Jharna Chakravorty, Johann Neumayer, Karsing Megu, Jaya Narah, Thomas Schmitt, Himender Bharti, Johannes Spaethe, Axel Brockmann

Supplementary material 2 Examples of bumble bee species collected in Arunachal Pradesh Data type: species data Explanation note: (A) Queens of the social parasite Bombus (Psithyrus) novus and two potential host species from the subgenus Melanobombus, (B) *B.miniatus* and (C) *B.rufofasciatus*. All species were found to co-occur in the west of Arunachal Pradesh (~ 4,200 m, Se-La Pass environment, Tawang Region). This dataset is made available under the Open Database License (http://opendatacommons.org/licenses/odbl/1.0/). The Open Database License (ODbL) is a license agreement intended to allow users to freely share, modify, and use this Dataset while maintaining this same freedom for others, provided that the original source and author(s) are credited.Martin Streinzer, Jharna Chakravorty, Johann Neumayer, Karsing Megu, Jaya Narah, Thomas Schmitt, Himender Bharti, Johannes Spaethe, Axel Brockmann
