# Supplementary figures and images for: Species composition and elevational distribution of bumble bees (Hymenoptera, Apidae, Bombus Latreille) in the East Himalaya, Arunachal Pradesh, India
Source: Zookeys. 2019 Jun 3;851:71–89. doi: 10.3897/zookeys.851.32956 (PMC6557907; doi:10.3897/zookeys.851.32956)

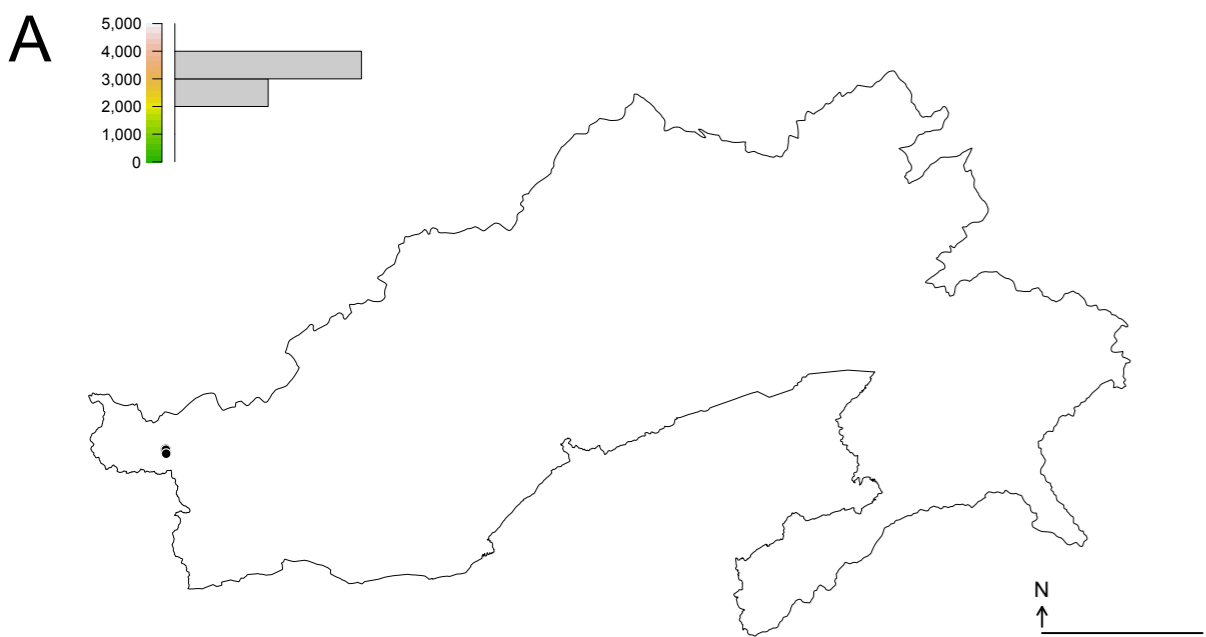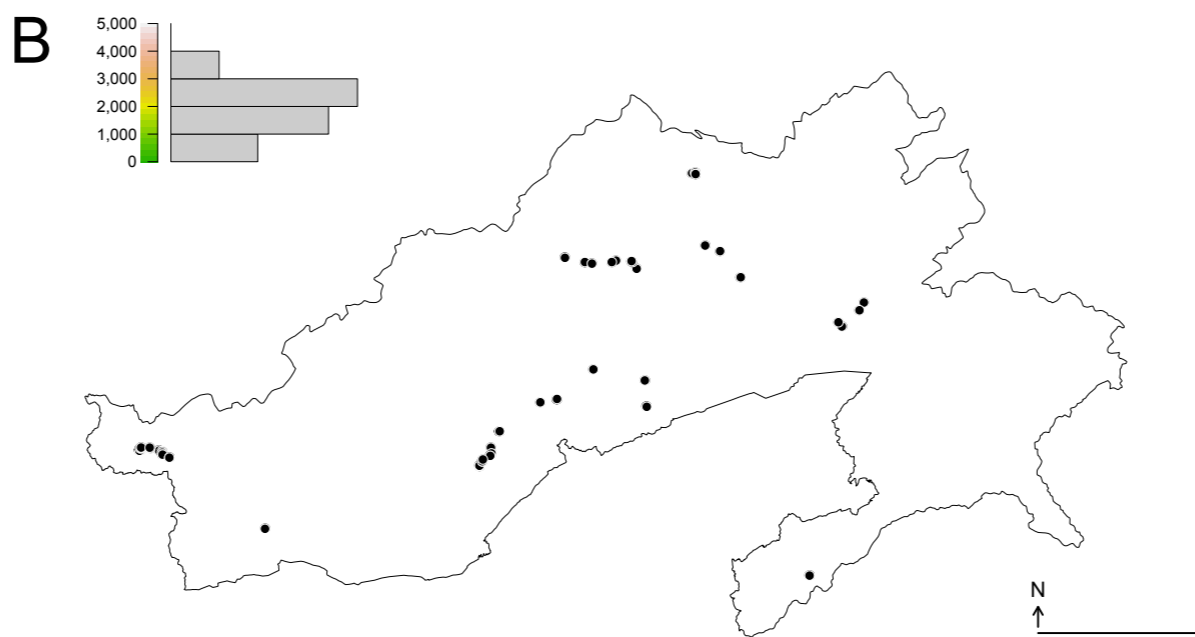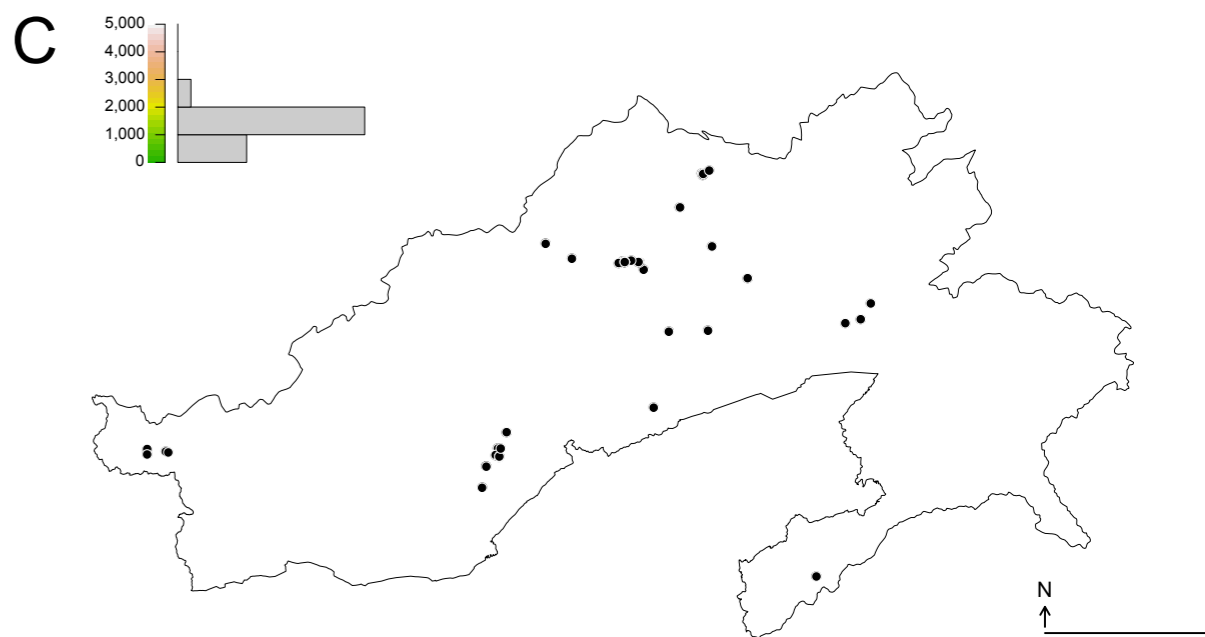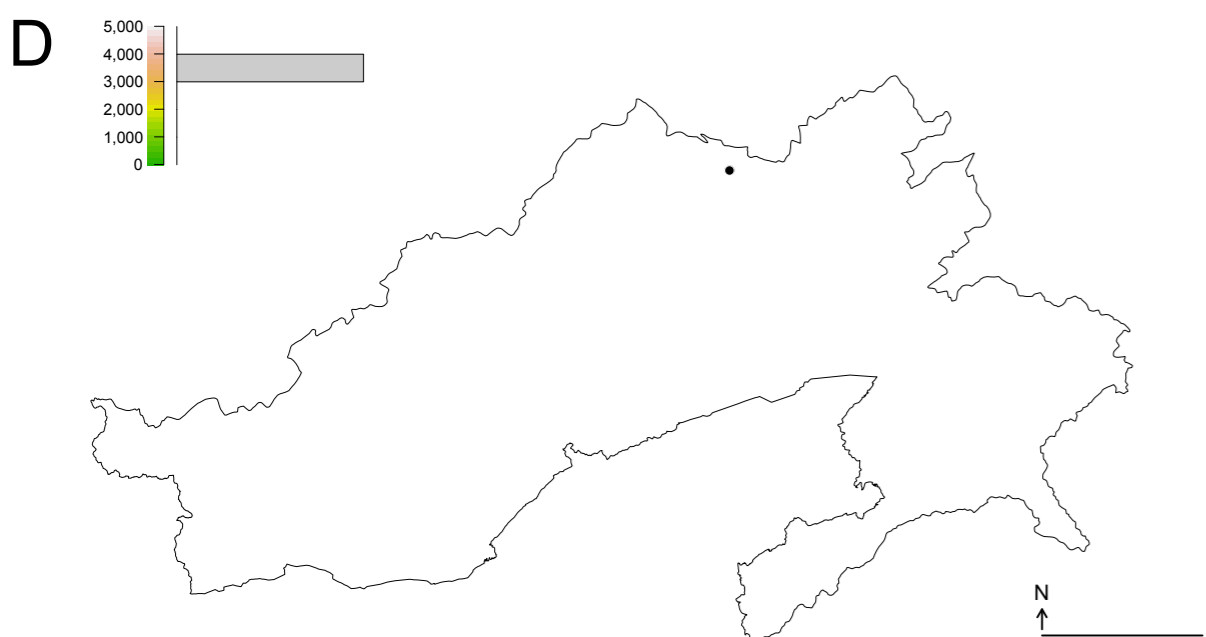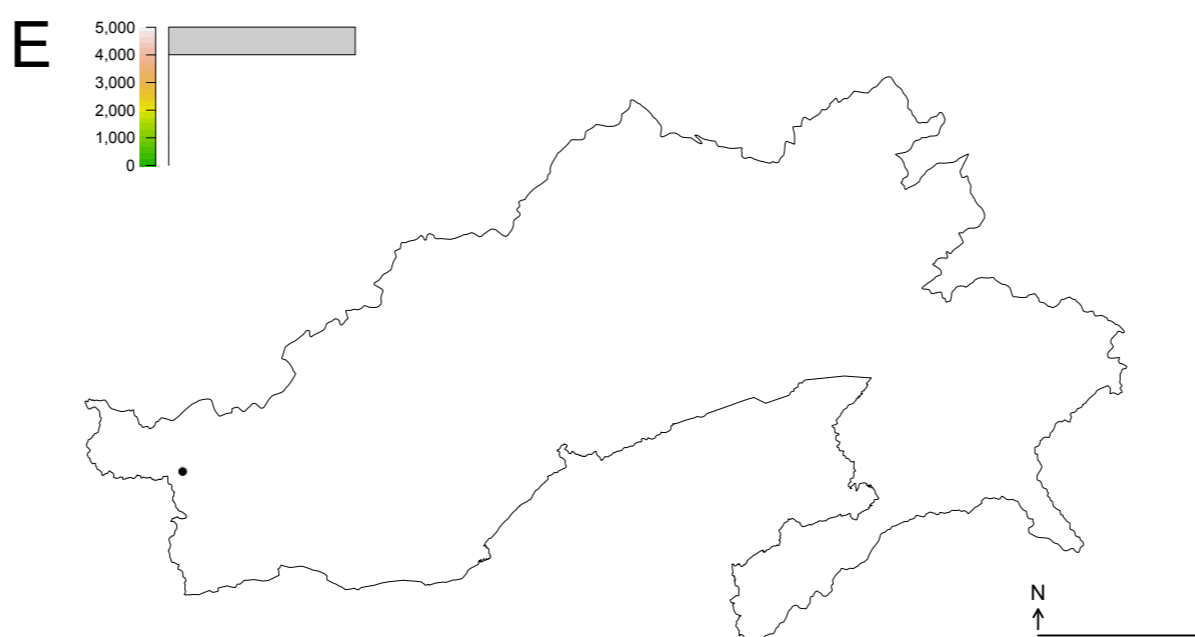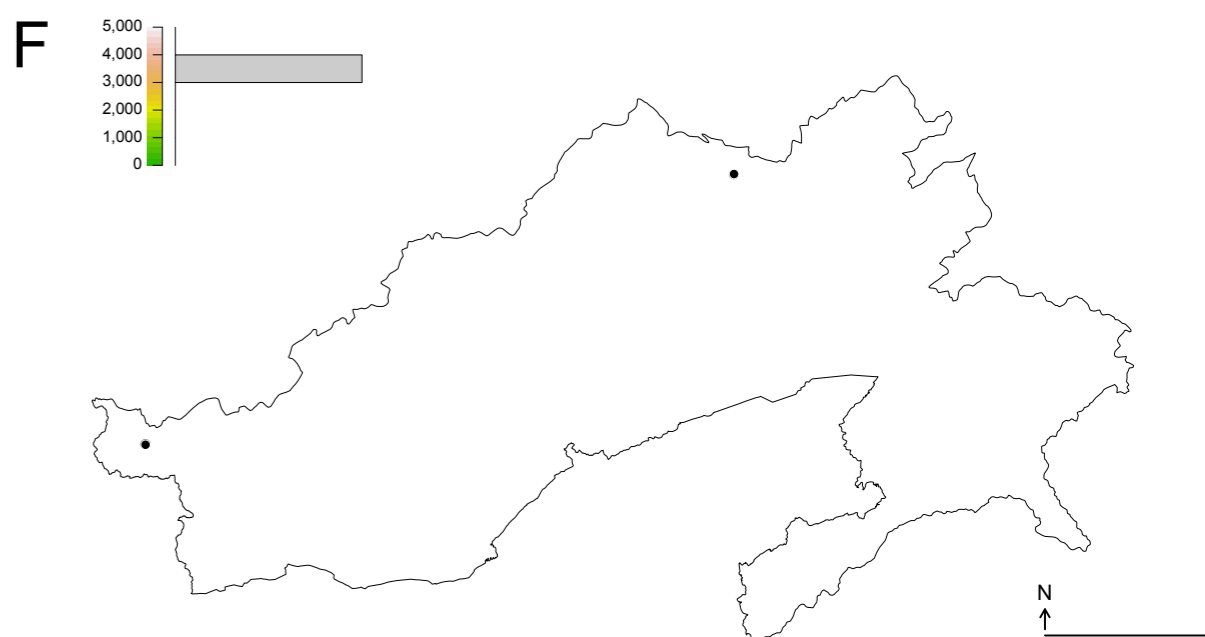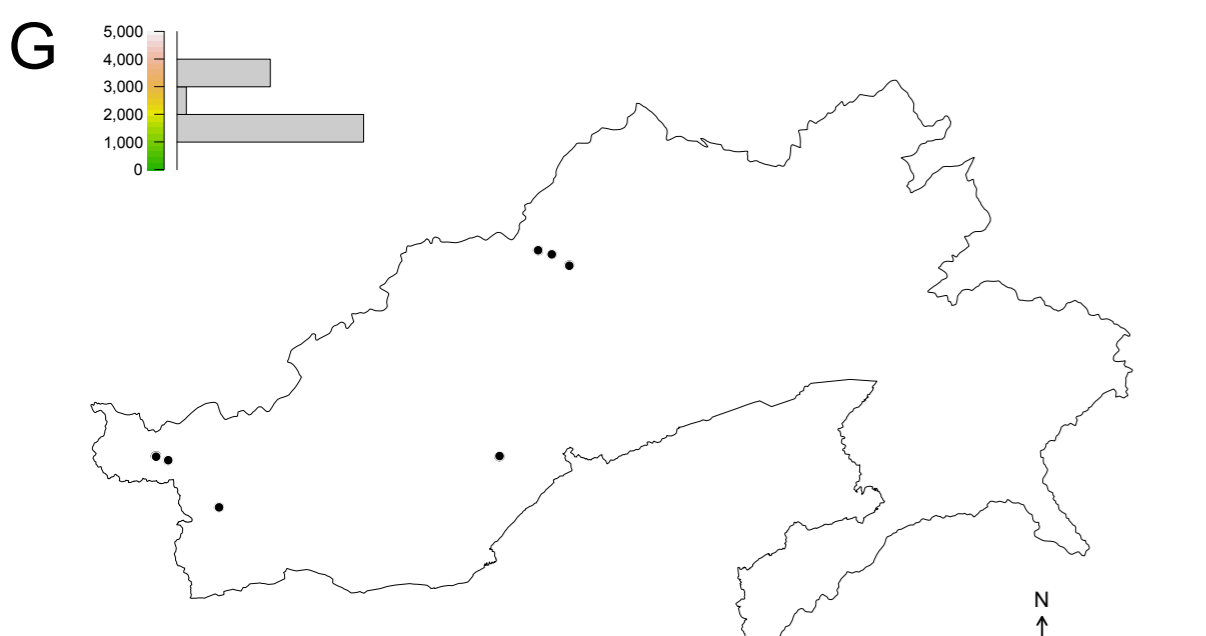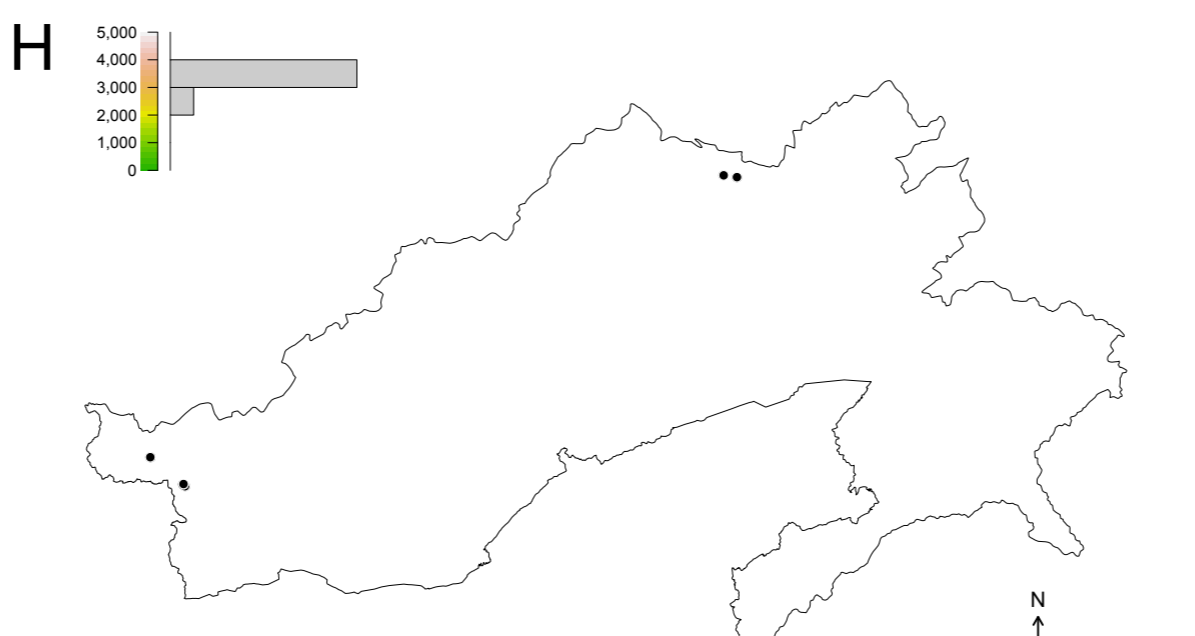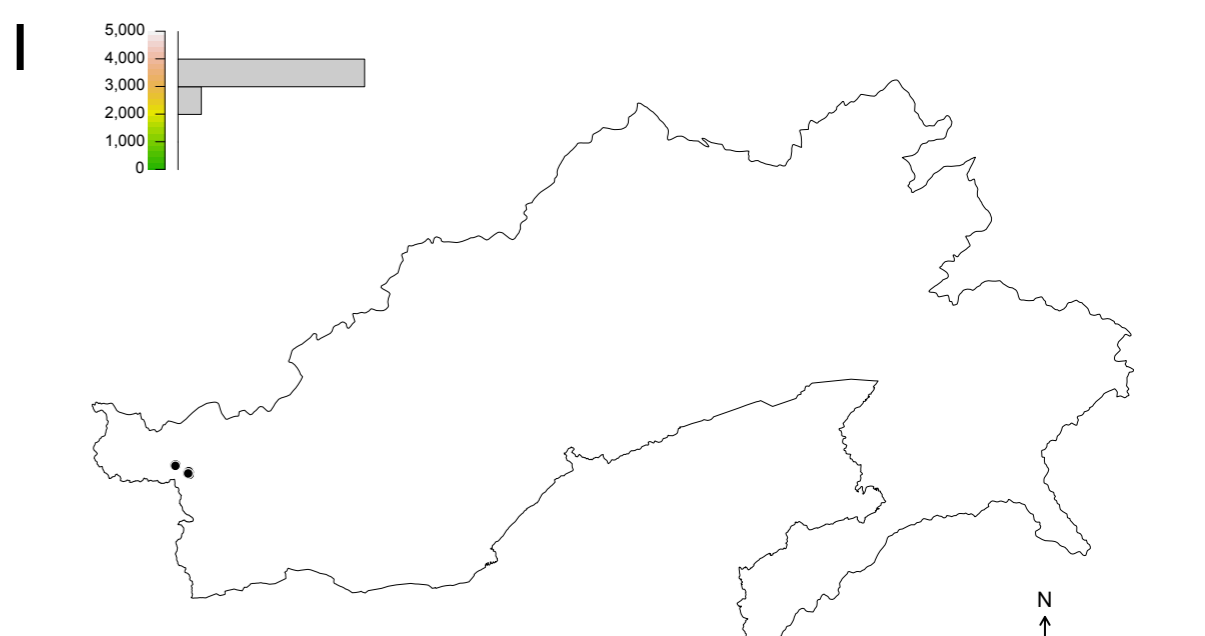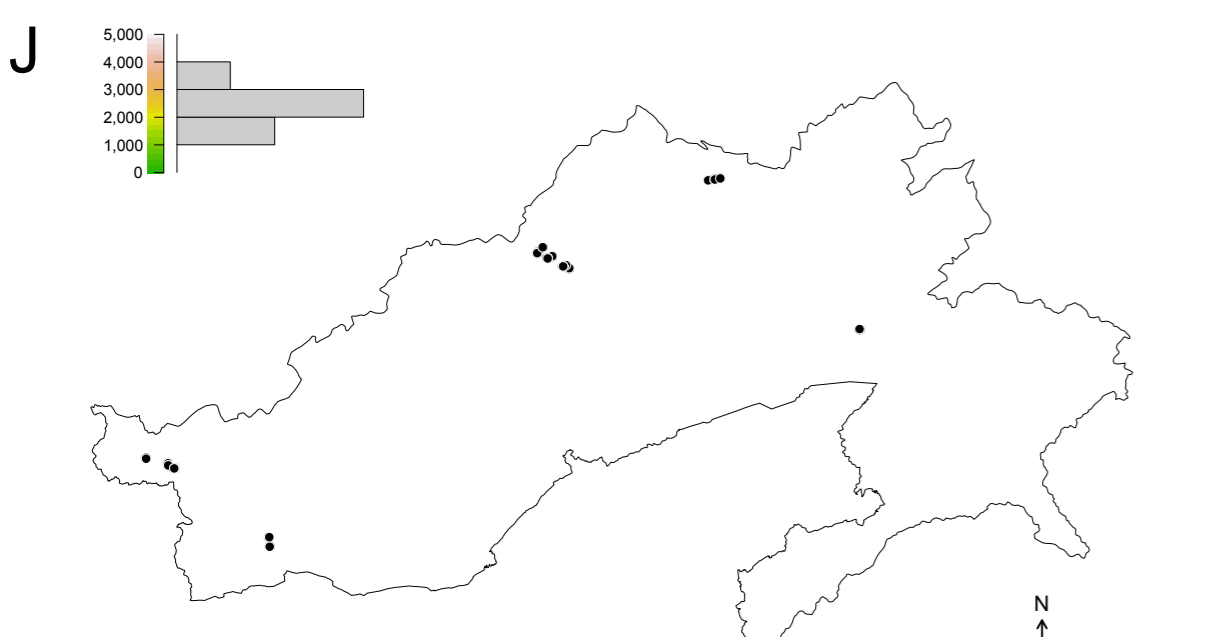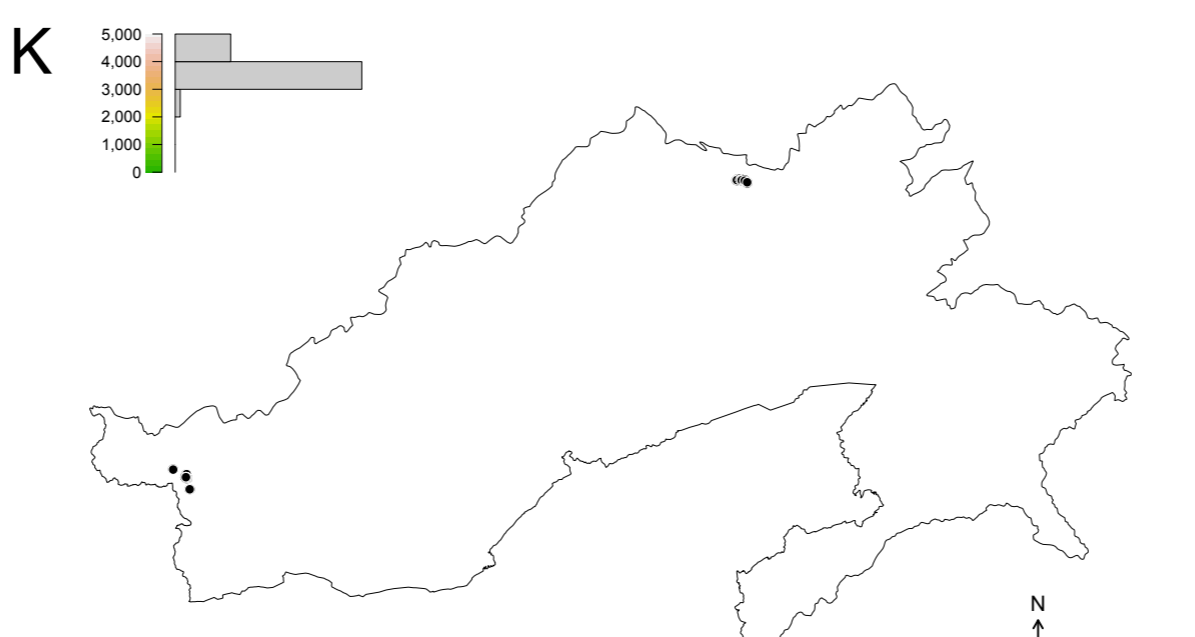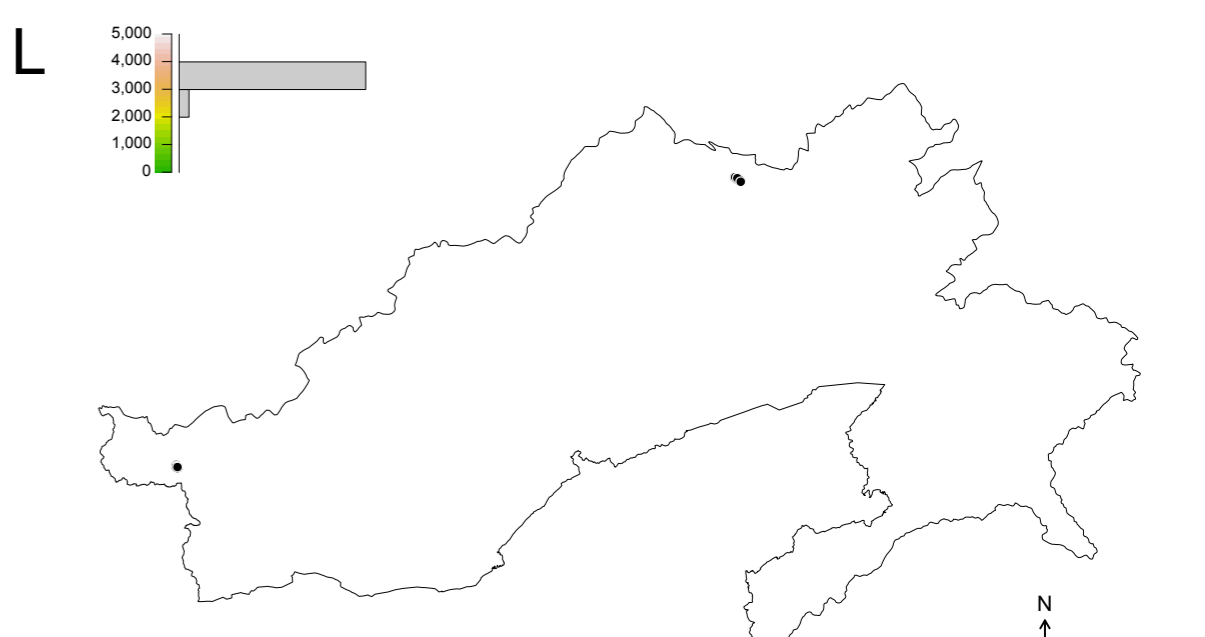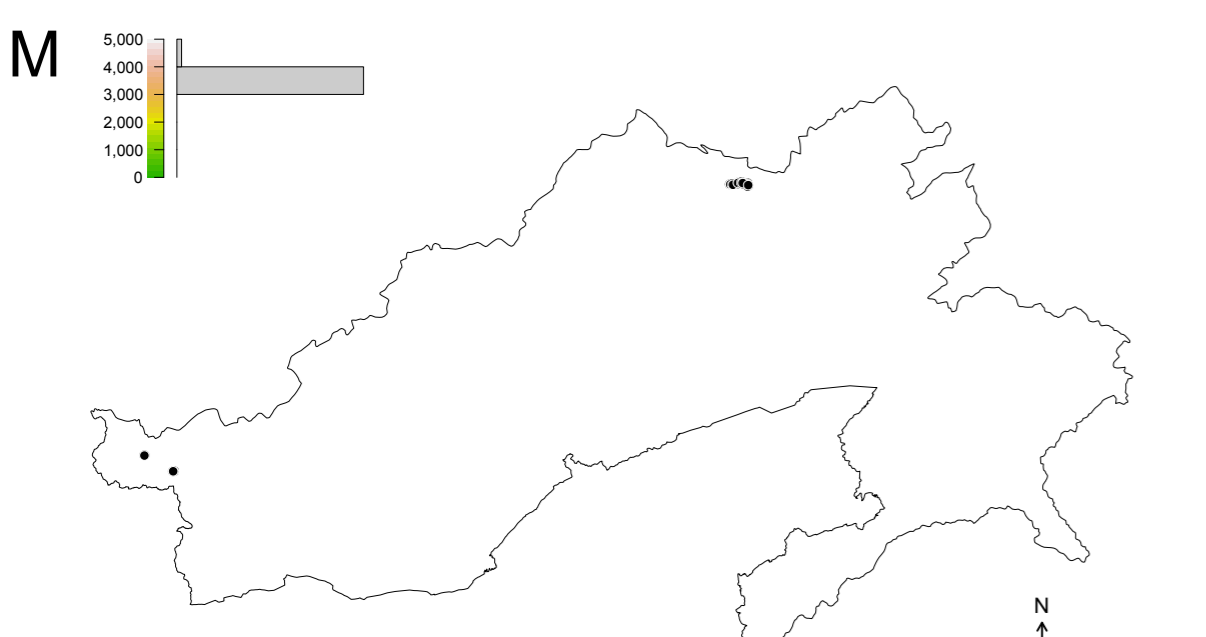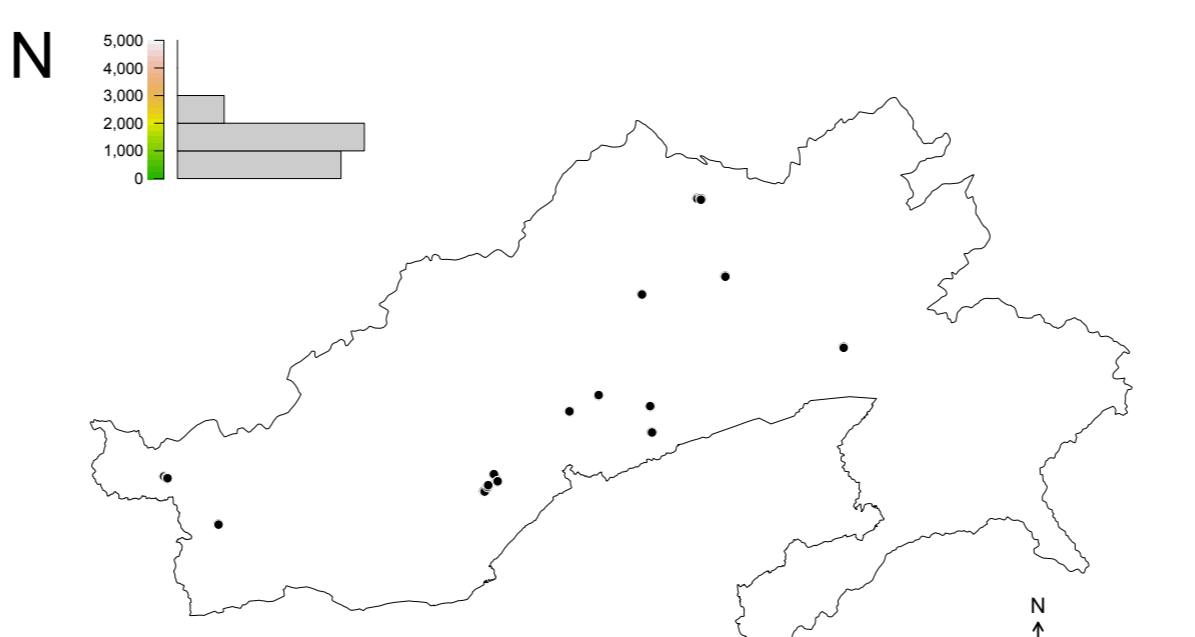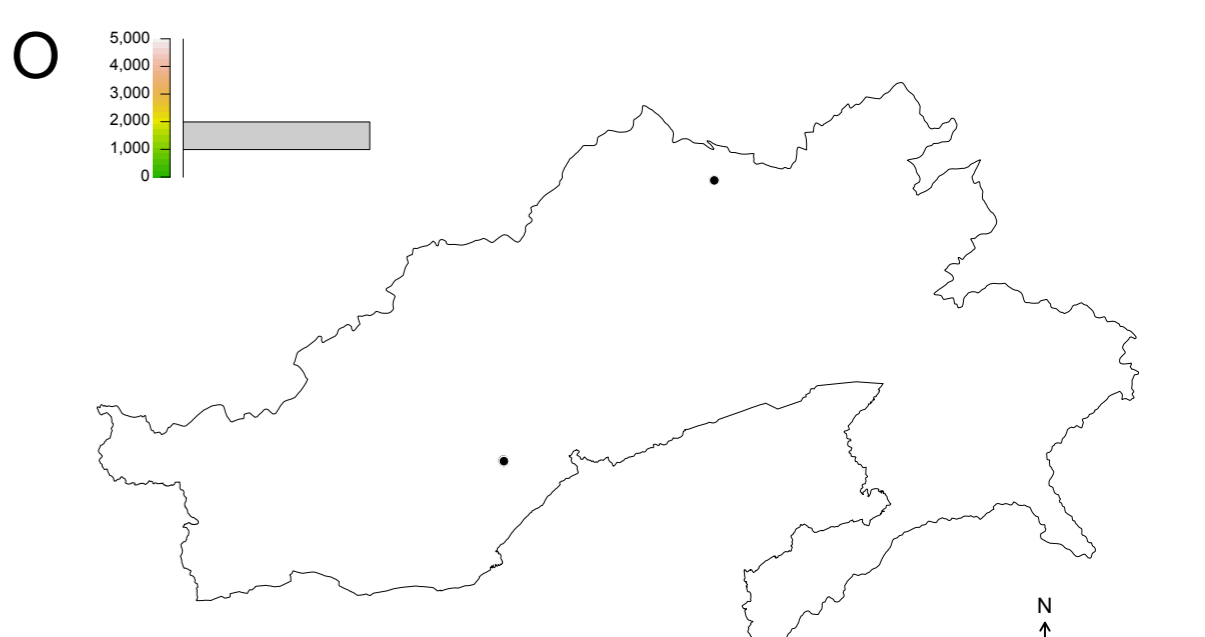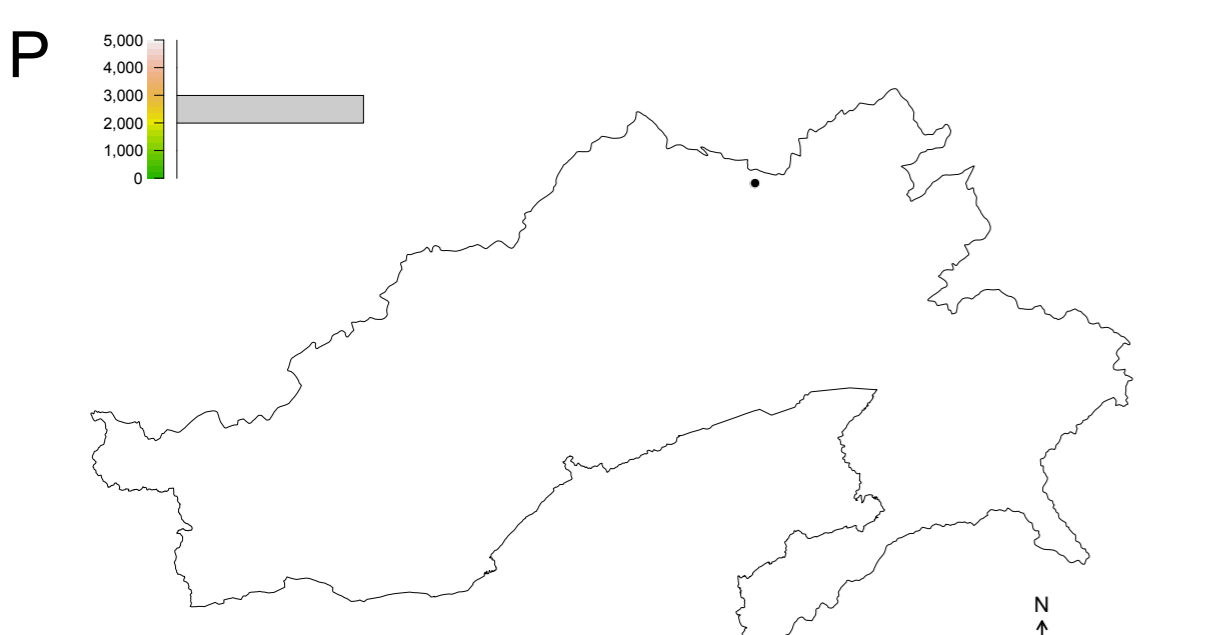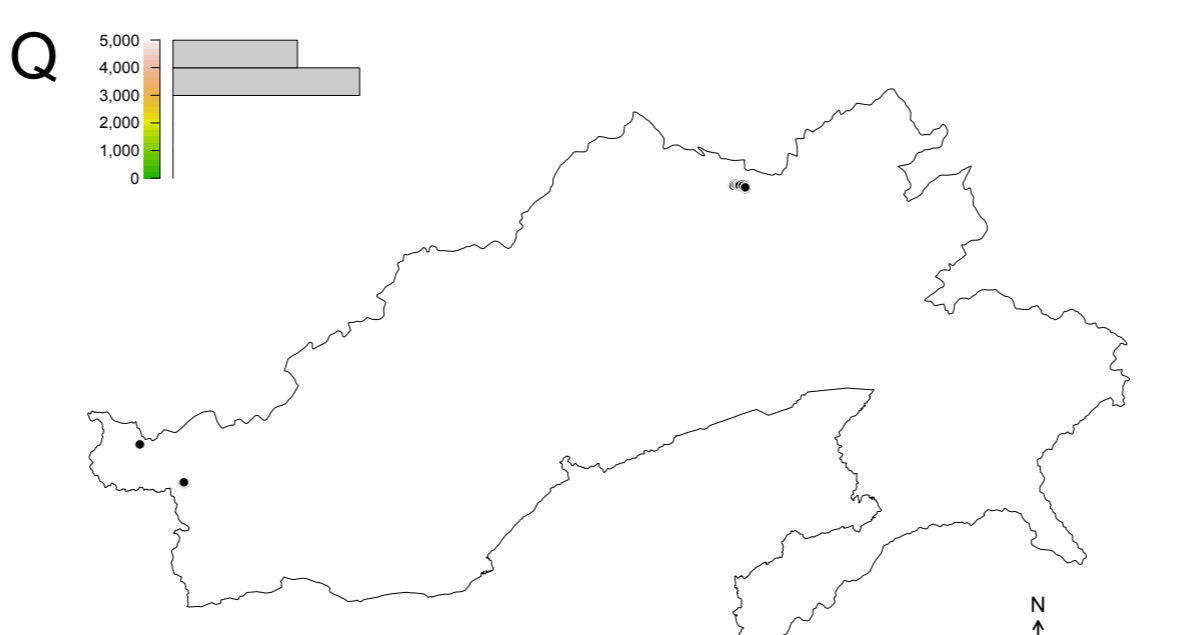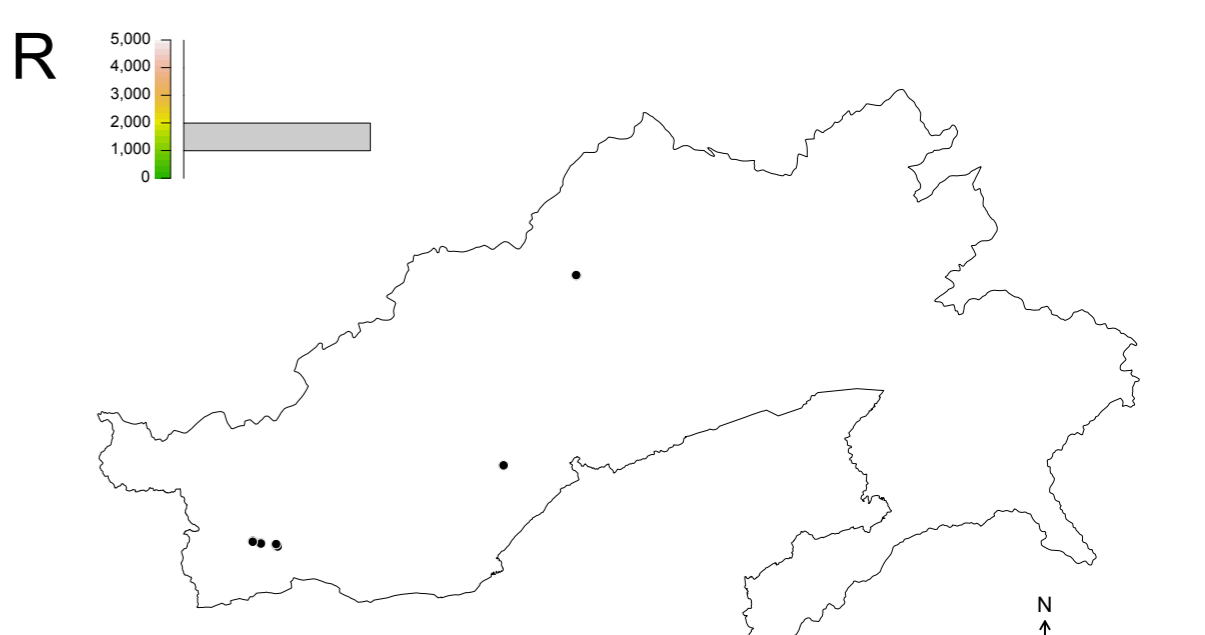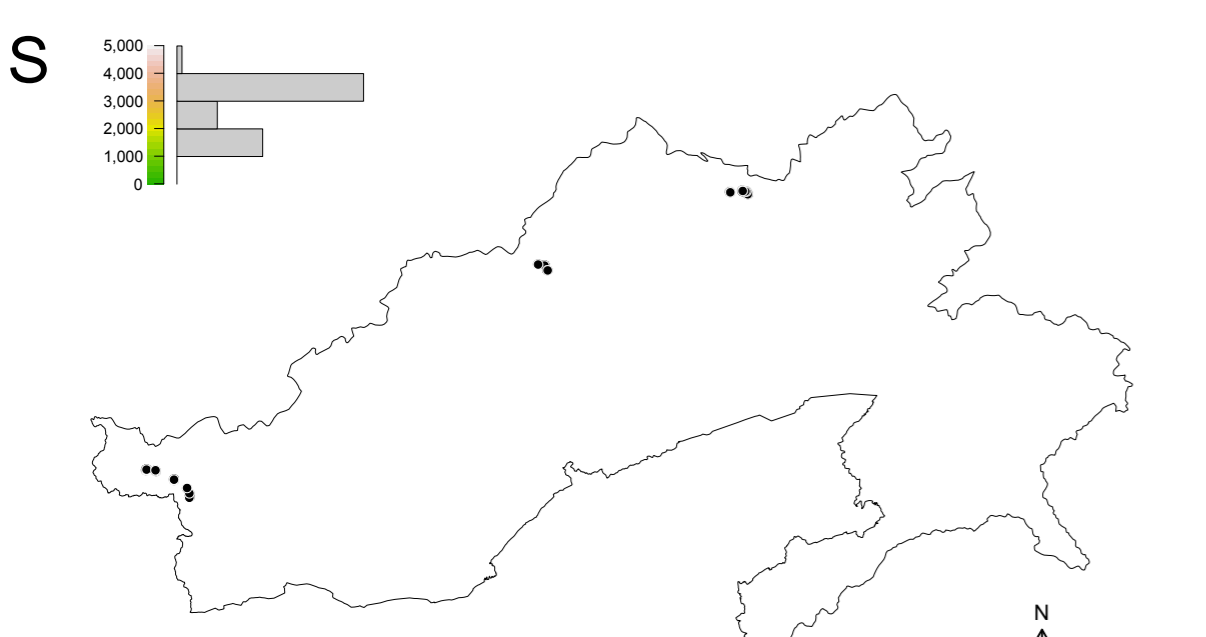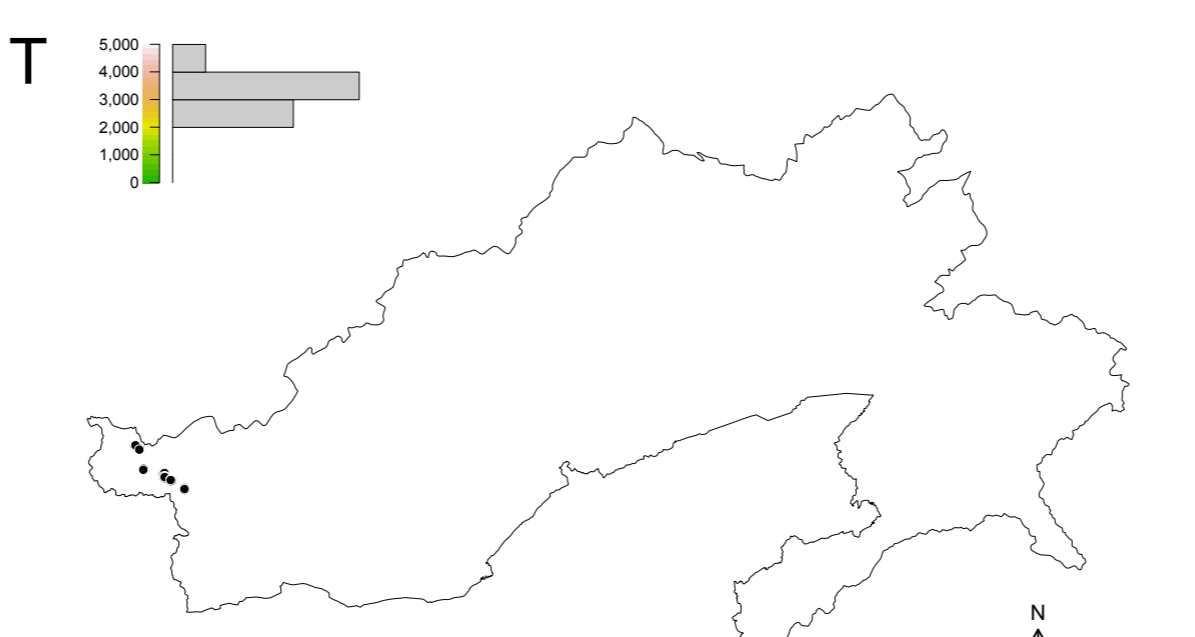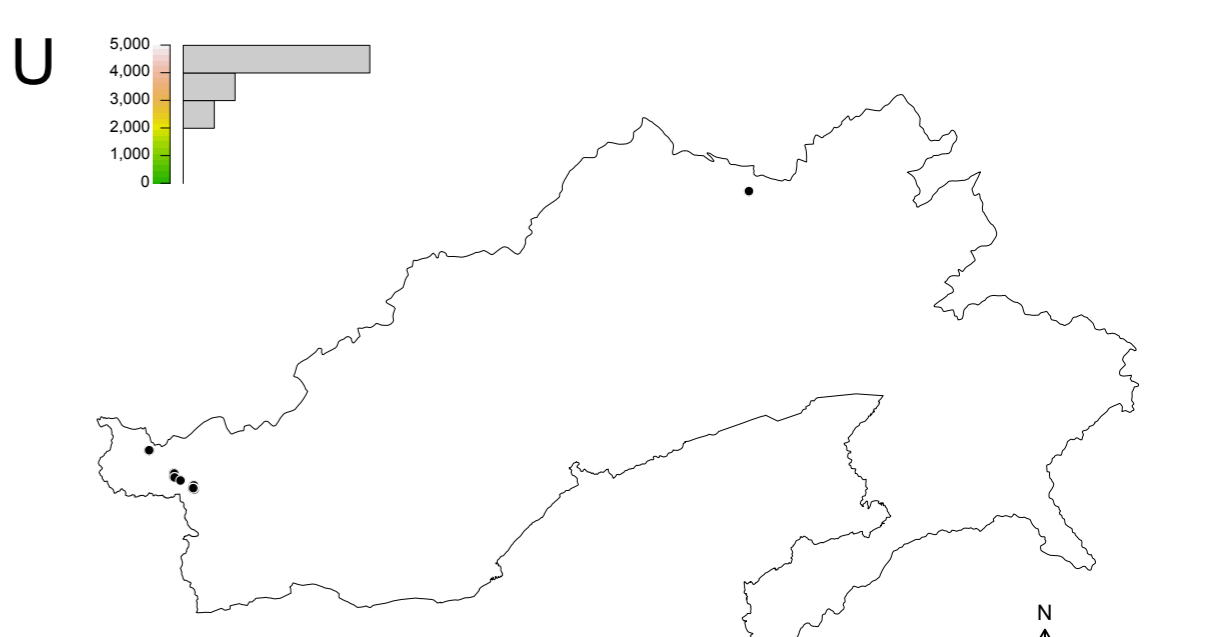

Supplement: Supplementary material 1 [file zookeys-851-071-s001.pdf]

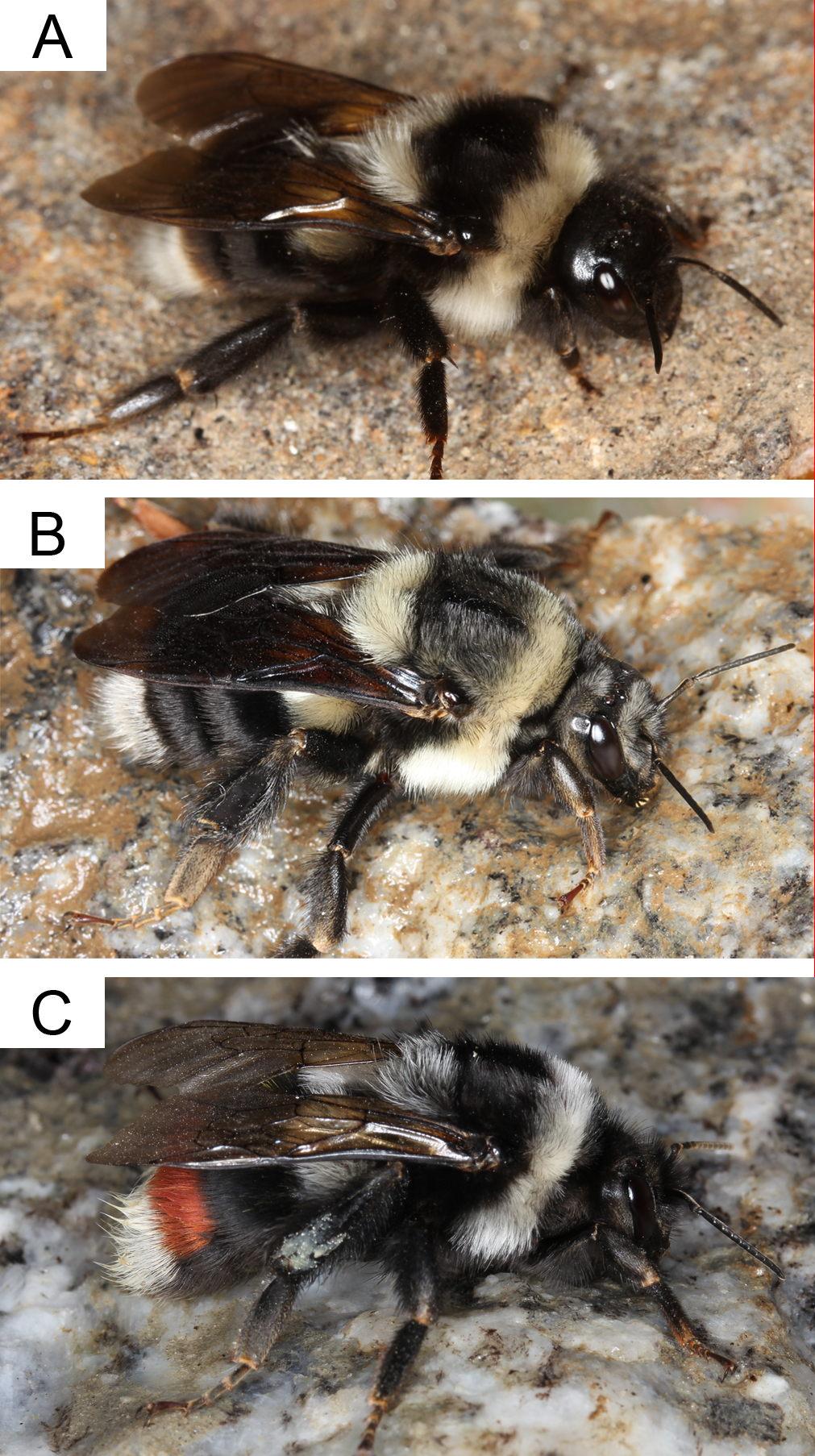

Supplement: Supplementary material 2 [file zookeys-851-071-s002.tif]
